# Ultrasound-assisted magnetic dispersive micro-solid-phase extraction based on carbon quantum dots/zeolite imidazolate framework-90/polyvinyl pyrrolidone/Fe_3_O_4_ followed by high-performance liquid chromatography with ultraviolet detection for trace analysis of paracetamol and etodolac in human plasma†

**DOI:** 10.1039/d4ra04875j

**Published:** 2024-10-22

**Authors:** Kimia Ahmadi, Aysan Abolfathi, Sana Nasirimoghadam, Negar Nasiri Moghaddam Kalkhoran, Mohsen Zeeb

**Affiliations:** a Department of Applied Chemistry, Faculty of Science, Islamic Azad University South Tehran Branch Tehran Iran zeeb.mohsen@gmail.com + 98 21 33722831

## Abstract

A four-part and sustainable nanocomposite composed of carbon quantum dots modified with zeolite imidazolate framework-90, polyvinyl pyrrolidone and magnetite (CQDs/ZIF-90/PVP/Fe_3_O_4_) was fabricated and applied in ultrasound-assisted magnetic dispersive micro-solid-phase extraction (US-A-MDMSPE). US-A-MDMSPE was followed by high-performance liquid chromatography with ultraviolet detection (HPLC-UV) for extraction, enrichment and simultaneous low-level monitoring of paracetamol (PCM) and etodolac (EDL) in human plasma. To increase the extraction yield and improve the sensitivity, nanohybrid arrays of metal–organic frameworks and conductive polymers were immobilized on the surface of CQDs followed by magnetization. The nano-extractor was characterized *via* Fourier transform infrared (FTIR) spectroscopy, X-ray diffraction (XRD), field-emission scanning electron microscopy (FE-SEM) and energy-dispersive X-ray spectroscopy (EDX). The recent method provided limits of detection (LODs) of 0.16 and 0.09 ng mL^−1^ for PCM and EDL, respectively. The calibration curve for PCM and EDL was linear in the range of 0.7–2000 ng mL^−1^ and 0.5–1200 ng mL^−1^ with a regression (*r*^2^) value between 0.993 and 0.998, respectively. Acceptable precisions including intra-assay (≤6.9%) and inter-assay (≤8.3%) accuracies and notable accuracy (≤8.5%) were achieved to demonstrate the applicability of this method for the evaluation of the pharmacokinetic data of target drugs in human plasma.

## Abstract 2

Schematic diagram of the synthesis routes of CQDs/ZIF-90/PVP/Fe_3_O_4_.

## Graphical abstract

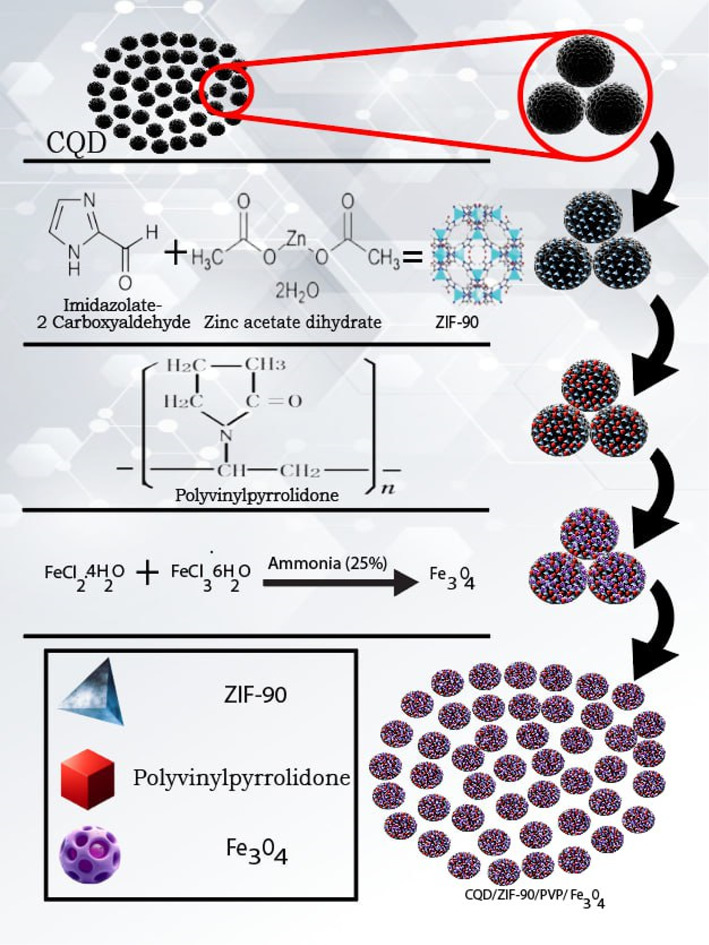

## 1. Introduction

**Fig. 1:**
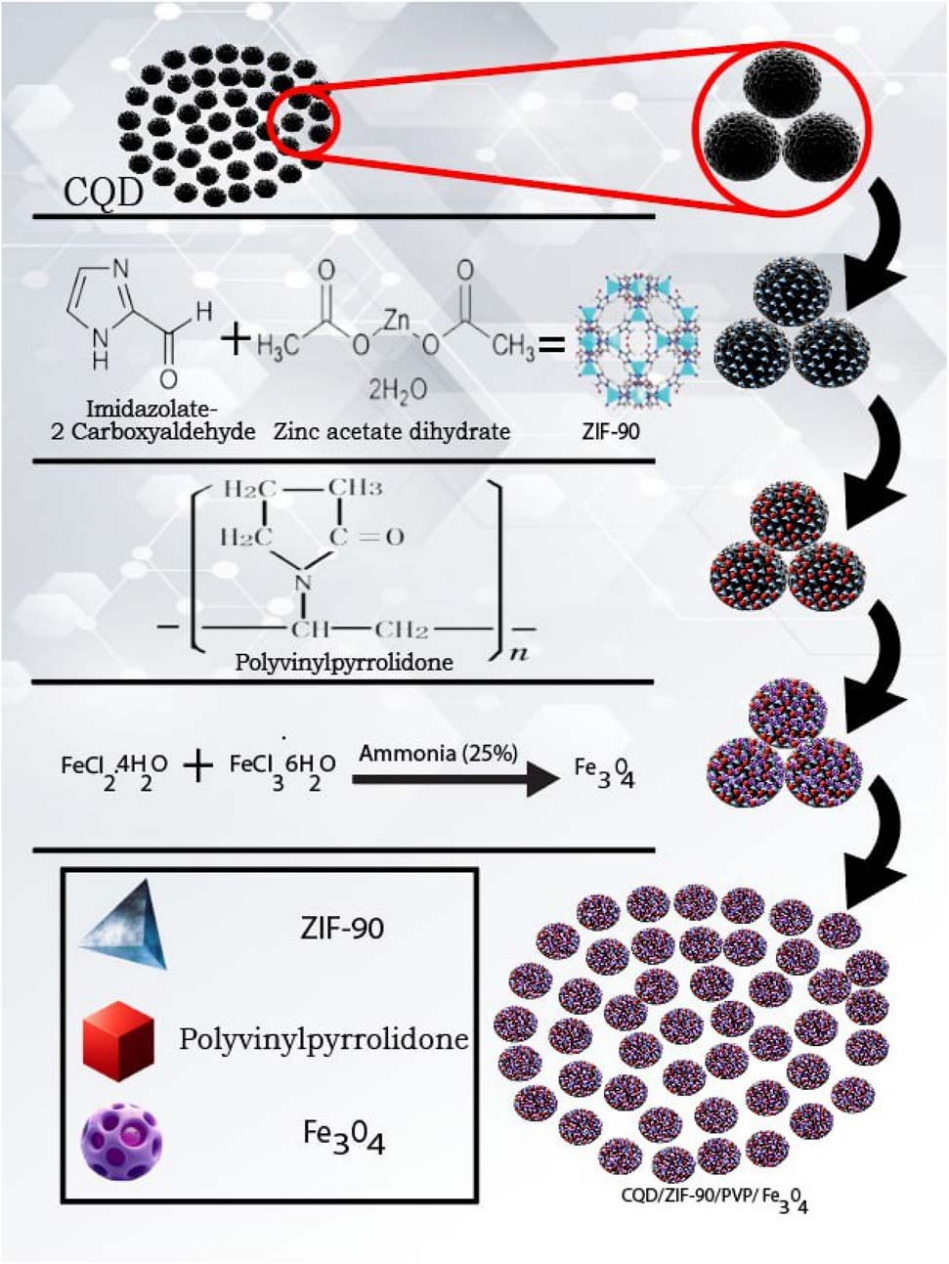
Schematic diagram of the synthesis routes of CQDs/ZIF-90/PVP/Fe_3_O_4_.

Non-steroidal anti-inflammatory drugs (NSAIDs) are used to cure inflammatory illnesses.^1^ Paracetamol (PCM) and Etodolac (EDL) are both categorized in this group.^2^ PCM, chemically known as *N*-(4-hydroxyphenyl)acetamide, has a molecular weight of 151.16 (Fig. 1SA†). PCM is widely used due to its analgesic and antipyretic effects, but it does not exhibit significant anti-inflammatory properties.^3,4^ EDL, with the chemical name 1,8-diethyl-1,3,4,9-tetrahydropyrano(3,4-*b*)-indole-1-acetic acid, has a molecular weight of 287.37 (Fig. 1SB†). Analgesic, anti-inflammatory and antipyretic properties are considered as the most effective features of EDL. In order to alleviate postoperation pain, inflammation, rheumatoid arthritis, and osteoarthritis, EDL is widely used.^5,6^

To improve the analgesic effects of PCM, it is preferred to be combined with other NSAIDs. As a result of this combination, the level of dose-dependent side effects of NSAIDs will be diminished.^7^ For instance, coupling of PCM with EDL in order to decrease osteoarthritis-associated severe pain can improve clinical consequences.^8^ PCM and EDL combinations are being recognized all around the world for a stronger and more effective remedial activity than that of either drug when used alone. Literature survey reveals that various analytical techniques have been used for the determination of PCM and EDL in different matrices. The most applicable techniques include liquid chromatography-tandem mass spectrometry (LC-MS/MS),^9^ high-performance liquid chromatography-ultraviolet detection (HPLC-UV),^10^ high-performance liquid chromatography-photodiode array detection (HPLC-PDA),^11^ gas chromatography-mass spectrometry (GC-MS),^12^ liquid chromatography-mass spectrometry (LC-MS),^13^ liquid chromatography-ion trap mass spectrometry^14^ and UV-Vis spectrophotometry.^15^

However, owing to matrix impact and low amounts of analytes in real samples, the application of robust sample preparation protocols prior to the measurement step is vital to meet the above-mentioned requirements. Among the sample enrichment protocols, ultrasound-assisted magnetic dispersive micro-solid-phase extraction (US-A-MDMSPE) offers some advantages including effective dispersion of sorbents through sample media during the utilization of ultrasound irradiation, higher mass transfer rate, reduction of toxic solvents, easy isolation of magnetic nanosorbents, and reduction of enrichment steps.^16–19^

Since sorbent plays special role in extraction method, development of new nano-extractors for providing reproducible and sensitive data is essential. Among new sorbents, carbon quantum dots (CQDs) as semi-conductor materials offer notable benefits including small size (10 nm), high surface area, high adsorption capability, water dispersibility, existence of oxygen-containing groups and hydrophobic–hydrophobic interactions between analytes and the sorbent.^20,21^ Due to the above-mentioned characteristics of these carbon-based materials, they have been widely applied in different multi-purpose analytical protocols.^22^ In order to increase the capability of carbon-based compounds for extraction, enrichment and isolation of target analytes, some crystalline structures such as metal–organic frameworks (MOFs) offer appropriate achievements for surface modification. Among MOFs, zeolitic imidazolate frameworks (ZIFs) are classified as novel MOFs with a three-dimensional structure in which metal centres are bonded with organic imidazolate (Im) binders.^23–26^

ZIF-90 has sample topology and pore entrance, and it has just a variation of 0.4 °A in its pore/cage capacities, and this type of MOF uses a 2-carboxaldehyde imidazolate linker with hydrophobic property.^27,28^ ZIF-90 reveals individual advantages such as high surface area, tuneable porosity and appropriate thermal steadiness, which make this type of material a suitable choice for fabricating hybrid multi-part sorbents. In the present study, ZIF-90 was applied for the modification of CQD surface to provide individual sorbents with enhanced properties.

The other superior alternatives for CQD modification are conductive polymers (CPs) including polypyrrole, polythionie, polyaniline and polythiophene.^29–32^ Due to the existence of conjugated bonds and π-systems in these types of polymers, their application as modifiers reveals notable improvement in CQD properties for the isolation and extraction of target analytes. Among CPs, polyvinyl pyrrolidone (PVP) is inert, non-toxic, temperature-resistant, pH-stable, biocompatible and biodegradable. In addition, PVP is a highly potential material and an easily constructible compound that can be considered as a superior and cost-effective surface modifier of CQDs, and as a conductive polymer, it enhances π–π interactions.^33,34^ Furthermore, Fe_3_O_4_ particles impart magnetic properties to the sorbent. These combined benefits enhance the extraction yield, making it easier to isolate the sorbent and improve the analytical performance.

In this work, nanoarrays of ZIF-90/PVP/Fe_3_O_4_ were chosen for improving the characteristics of CQDs to lead to the fabrication of a sustainable and promising hybrid sorbent with special abilities such as higher extraction yield, easy recyclability, and better reproducibility in data. The new designed sorbent was applied in the US-A-MDMSPE protocol, which was followed by HPLC-UV for simultaneous separation and quantification of PCM and EDL in human plasma. The applicability of the proposed method was evaluated by defining the main pharmacokinetic data including the half-life (*T*_1/2_), the time of reaching the maximum concentration (*T*_max_), the maximum plasma concentration (*C*_max_), area under the curve (AUC_0–12_) and area under the curve at infinite time (AUC_0–∞_).

## 2. Experimental

### 2.1. Chemicals

In this work, the analytical grade chemicals were used without further purification. These chemicals including citric acid, imidazolate-2-carboxyaldehyde, dimethyl sulfoxide, Zn(CH_3_COOH)_2_·2H_2_O, *N*,*N*-dimethylformamide, polyvinylpyrrolidone, graphite powder, iron(iii) chloride hexahydrate (FeCl_3_·6H_2_O), iron(ii) chloride tetrahydrate (FeCl_2_·4H_2_O), ethanol (C_2_H_5_OH), dimethyl sulfoxide (DMSO), dimethylformamide (DMF), chloroform, urea (CH_4_N_2_O), ammonia solution 25% (NH_3_) and acetonitrile (ACN) were obtained from Merck Company (Darmstadt, Germany). The standard drugs of both PCM (purity ≥ 97%) and EDL (purity ≥ 98%) were obtained from Darupakhsh Company (Tehran, Iran). A tablet containing a combined dose of 500 mg PCM and 400 mg EDL was bought from commercial sources. Furthermore, HPLC-grade methanol, acetonitrile and potassium dihydrogen phosphate were bought from Merck (Darmstadt, Germany). Fresh blood samples were provided by Iranian Blood Transfusion Organization (Tehran, Iran). All the plasma samples were stored at −18 °C until use.

### 2.2. Instrumentation and conditions

Fourier transform-infrared (FT-IR) spectra were recorded using A Vector 22 FT-IR spectrometer (Bruker, Germany). Field-emission scanning electron microscopic (FE-SEM) images were acquired using a Mira 3-XMU (Tescan, Czech Republic). X-ray diffraction (XRD) spectra were recorded using an D8 Advance instrument (Bruke, Germany) with Cu Kα radiation (*λ* = 1.54 A°). Energy-dispersive X-ray (EDX) spectra were recorded using a Tescam-Vega3 (Czech Republic) and a Zeiss Sigma Vp machine (Jena, Germany).

### 2.3. HPLC assessment

All chromatograms were obtained using an HPLC instrument (Waters Alliance e2695, Massachusetts, USA) under the following conditions: dual-wavelength detector model waters 2487, C_18_ column (Luna 5 μm C_18_ 100A HPLC column 250 × 4.6 mm id, Phenomenex Co, Torrance, CA) and an operation temperature of 30 °C. In order to record chromatograms, the wavelength was set at 230 nm and the volume of injection was tuned at 20 μL. For the elution process, isocratic condition was selected with the following details: acetonitrile–water (at pH 4.5 using a phosphate buffer) with a volume ratio of 65/35 V/V was used as the mobile phase at a flow rate of 1 mL min^−1^. For the removal of impurities, the mobile phase was filtered through a 0.2 μm polytetrafluoroethylene (PTFE) (Millipore, Bedford, MA, USA) membrane filter and the applied mobile phase was degassed before the analysis.

### 2.4. Synthesis of CQDs

For the synthesis of CQDs, a hydrothermal procedure was applied as follows: first, 3 g citric acid and 1 g urea were mixed and 20 mL deionized water was added to them and stirred for 5 min at a temperature of 25 °C. Then, the above solution was heated in an oven at 200 °C for 7 h. Finally, the obtained product was cooled to room temperature to obtain a black powder.^35^ Using the mass of final product and calculating its number of moles and according to the molar ratio of initial material, the synthesis yield was calculated.

### 2.5. Synthesis of ZIF-90

For the preparation of ZIF-90, 10 mL of imidazolate-2-carboxyaldehyde in DMSO was added to 10 mL (0.2 M) Zn(CH_3_COOH)_2_·2H_2_O in DMF and stirred for 15 min. After this step, another 20 mL DMF was added into the above solution, and it was stirred for another 5 min. The remaining material was obtained, washed two times with ethanol and was dried under vacuum for 12 h.^36^ The yield of reaction was calculated using the number of moles of initial materials and final materials.

### 2.6. Surface modification of CQDs with ZIF-90

For the fabrication of CQDs/ZIF-90, 0.2 g CQDs and 0.2 g ZIF-90 were mixed and dissolved in ethanol and deionized water (50 : 50 V/V). In order to provide a suitable suspension, the obtained solution was sonicated in an ultrasound bath for 5 min, at a frequency of 25 kHz and a power of 60 W. The solution was kept into an oven at a temperature of 100 °C for 24 h to obtain CQDs/ZIF-90.^37^

### 2.7. Fabrication of CQDs/ZIF-90/PVP

For the preparation of CQDs/ZIF-90/PVP, 0.2 g of CQDs/ZIF-90 and 0.2 g of polyvinylpyrrolidone were dispersed in ethanol and deionized water (50 : 50 V/V). Then, the solution was kept in an ultrasound bath for 10 min. CQDs/ZIF-90/PVP was placed in an oven at a temperature of 100 °C and then the water content was removed. The resulting product was kept in an evaporating dish and dried in a hot air oven at 150 °C overnight.

### 2.8. Fabrication of CQDs/ZIF-90/PVP/Fe_3_O_4_

The fabrication of Fe_3_O_4_ nanoparticles was carried out based on the following procedure: Briefly, 0.246 g FeCl_3_·6H_2_O and 0.096 g of FeCl_2_·4H_2_O were dissolved in 22.5 mL double-distilled water and then 0.093 g of CQDs/ZIF-90/PVP was added to the mentioned solution under a flow of nitrogen gas as an inert medium. In the next step, the solution was stirred for one hour at room temperature. After that, the ammonia solution with a concentration of 25%. V/V was added dropwise to the mixture to fix the pH value at 10 and then the temperature was increased to 80 °C and stirred for an extra hour while nitrogen was still flowing. At the end, the magnetic product was separated from the media, washed with distilled water and dried in an oven under vacuum at 80 °C for 2 h. All the synthesis routes are shown in Fig. 1.

### 2.9. Deproteinization of plasma

One of the most important steps prior to human plasma analysis is the protein precipitation of blood to reduce the interferents.^38,39^ For this process, human blood samples were transferred in the tubes containing ethylenediaminetetraacetic acid (EDTA), centrifuged and stored at −18 °C until use. For the preparation of spiked human plasma, the frozen samples were thawed at room temperature (23 ± 0.5 °C) and the following procedure was performed: 1.9 mL of plasma sample was spiked with 100 μL of the standard solutions to achieve desirable concentrations of every target drug. After that, 2 mL of acetonitrile was added as the deproteinizing material and the sample was vortexed for 5 min and centrifuged for 6 min at 5000 rpm. In order to evaporate the acetonitrile content of sample, a flow of nitrogen was used. Ultimately, 4.0 mL buffer at pH 4.0 was added to the deproteinized sample and 5.0 mL of the sample was used for the extraction and enrichment of target drugs.

### 2.10. Working standard solutions, quality control samples and calibration curve plotting

In this study, spiked samples were used as standard solutions to reduce the effect of interfering compounds in the determination of analytes. First, 1.9 mL of plasma sample was spiked with 100 μL of standard solution of target drugs, to obtain desirable concentrations within the dynamic range. Then, 100 mg L^−1^ stock solutions of PCM and EDL were provided weekly by dissolving a required amount of each drug in HPLC-grade methanol. For the preparation of working solutions, the prepared stock solutions were diluted with deionized water to provide desirable levels of each drug. Plasma samples were spiked with working solutions for use as standard solutions. The dynamic linear range was evaluated and the calibration curve was plotted using spiked plasma samples. The quality control (QC) samples at concentration levels of 10, 250, 500, and 1500 ng mL^−1^ were prepared and stored at −18 °C. Different amounts of target drugs from a low concentration to a high concentration were checked and the linear ranges with *r*^2^ above 0.990 were defined. The QC samples were used to investigate the precision and accuracy of the present method.^40,41^

### 2.11. Ultrasound-assisted magnetic dispersive micro-solid-phase extraction followed by HPLC-UV (US-A-MDMSPE-HPLC-UV)

**Fig. 2:**
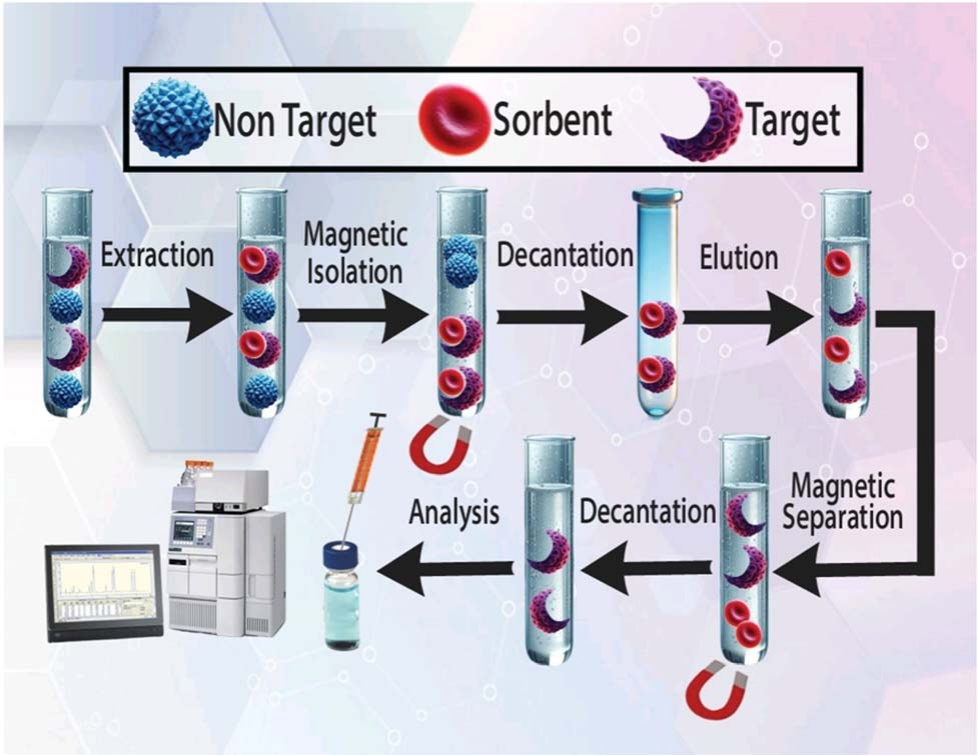
Various steps of the US-A-MDMSPE-HPLC-UV process for the isolation, enrichment and quantification of PCM and EDL.

For sample pretreatment, 5.0 mL of the prepared plasma sample at pH 4.0 was placed in a sample tube. After that, 30.0 mg of CQD/ZIF-90/PVP/Fe_3_O_4_ was added and the sample was sonicated for 5.0 min to disperse the extractor throughout the aqueous media. After dispersion process, drugs under study were extracted and loaded on the surface of the nanosorbent. After that, the sample tube was exposed to a powerful magnet (Nd-Fe-B-Nd, 0.8 tesla) to separate the nanosorbent from media. The aqueous media were removed using a pipette and the loaded nanosorbent was washed with 2.0 mL acetonitrile while sonication was performed within 4 min. In the final step, the tube was exposed to a magnet to collect the magnetic sorbent, and the desorbing solvent was evaporated using a nitrogen stream during heating. For the quantification of PCM and EDL the remaining material was dissolved in 50 μL of mobile phase of HPLC and 20 μL of that was injected into the column. The schematic diagram of all extraction, enrichment and quantification steps is shown in Fig. 2.

## 3. Results and discussion

### 3.1. Characterization

**Fig. 3:**
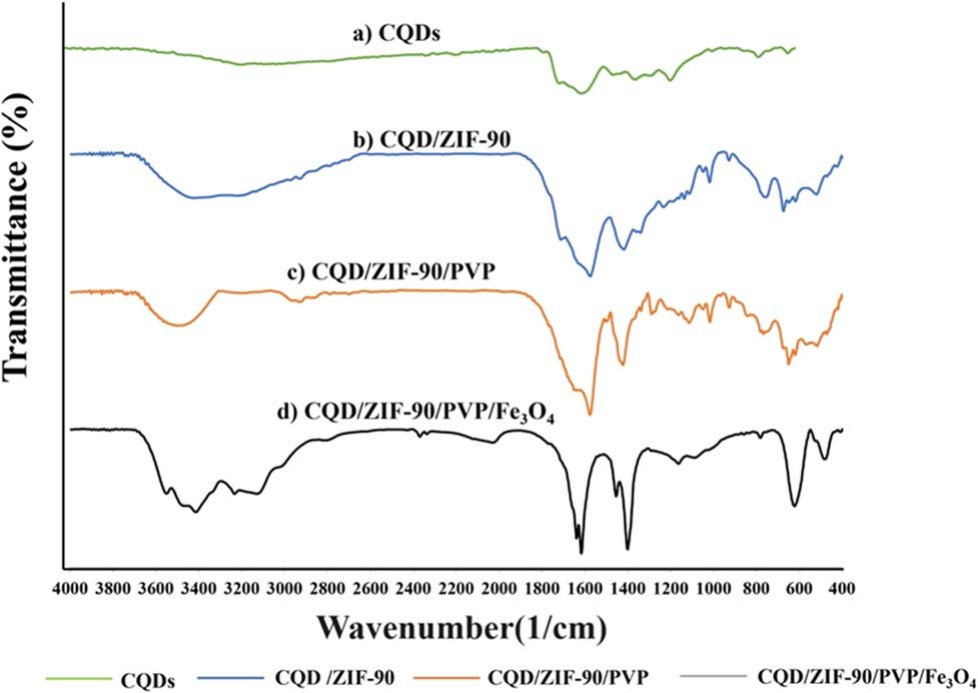
Fourier transform infrared spectra of (a) CQDs, (b) CQDs/ZIF-90, (c) CQDs/ZIF-90/PVP and (d) CQDs/ZIF-90/PVP/Fe_3_O_4_.

The fabricated CQDs/ZIF-90/PVP/Fe_3_O_4_ was evaluated and characterized by FTIR spectroscopy, XRD, EDX spectroscopy and FE-SEM. Fig. 3 shows the FTIR spectra of CQDs, CQDs/ZIF-90, CQDs/ZIF-90/PVP and CQDs/ZIF-90/PVP/Fe_3_O_4_. The FTIR spectrum of CQDs exhibits a relatively broad and weak peak around 3300 cm^−1^, which is related to hydroxyl groups in the structure of CQDs. Furthermore, the peaks located at 1610 cm^−1^ and 1405 cm^−1^ are assigned to C=O and C=C, respectively.^42^ C–O epoxy exhibits stretching vibration and shows a peak around 1130 cm^−1^ (Fig. 3a).^17^ As shown in Fig. 3b (CQDs/ZIF-90), the peak located at 1670 cm^−1^ corresponds to the imine (C=N) bond stretching. The peaks at 3400 to 3500 cm^−1^ are assigned to the secondary amine N–H bonds. The weak bond at 1290 cm^−1^ is assigned to the C–N stretching vibration of the imidazole ring. In addition, the peak around 1400 cm^−1^ can be referred to the C=N bond vibration. Furthermore, the vibration peaks in the range of 900–1400 cm^−1^ and the peaks below 800 cm^−1^ are assigned to in-plane bending and out-of-plane bending of the imidazole ring.^37^ All the obtained data reveal the prosperous modification of the CQD surface with ZIF-90. CQDs/ZIF-90/PVP (Fig. 3c) exhibits a peak around 1700 cm^−1^ which is assigned to the C=O stretching vibration caused *via* interaction between CQDs and existing rings in the polymer *via* π–π interactions and hydrogen bonding.^43^Fig. 3b shows the FTIR spectrum of CQDs/ZIF-90/PVP/Fe_3_O_4_, and in this spectrum, the peak located at 600 cm^−1^ reveals the stretching vibration of Fe–O, demonstrating the proper modification of CQDs/ZIF-90/PVP with magnetite particles.^18^

**Fig. 4:**
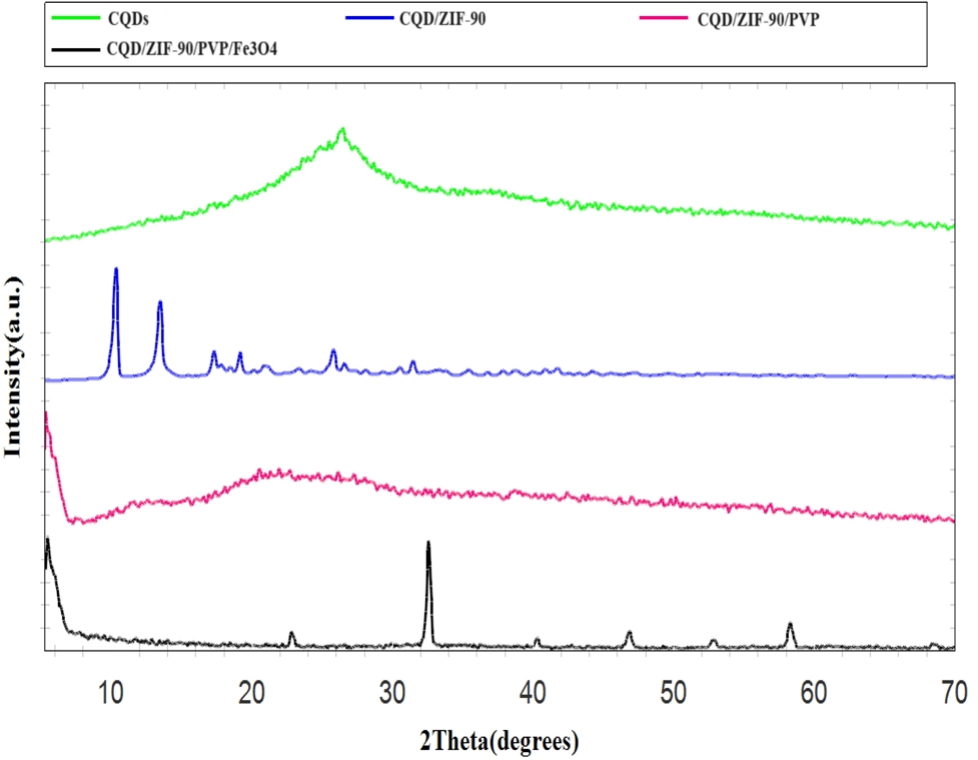
X-ray diffraction patterns of CQDs, CQDs/ZIF-90, CQDs/ZIF-90/PVP and CQDs/ZIF-90/PVP/Fe_3_O_4_.

**Fig. 5:**
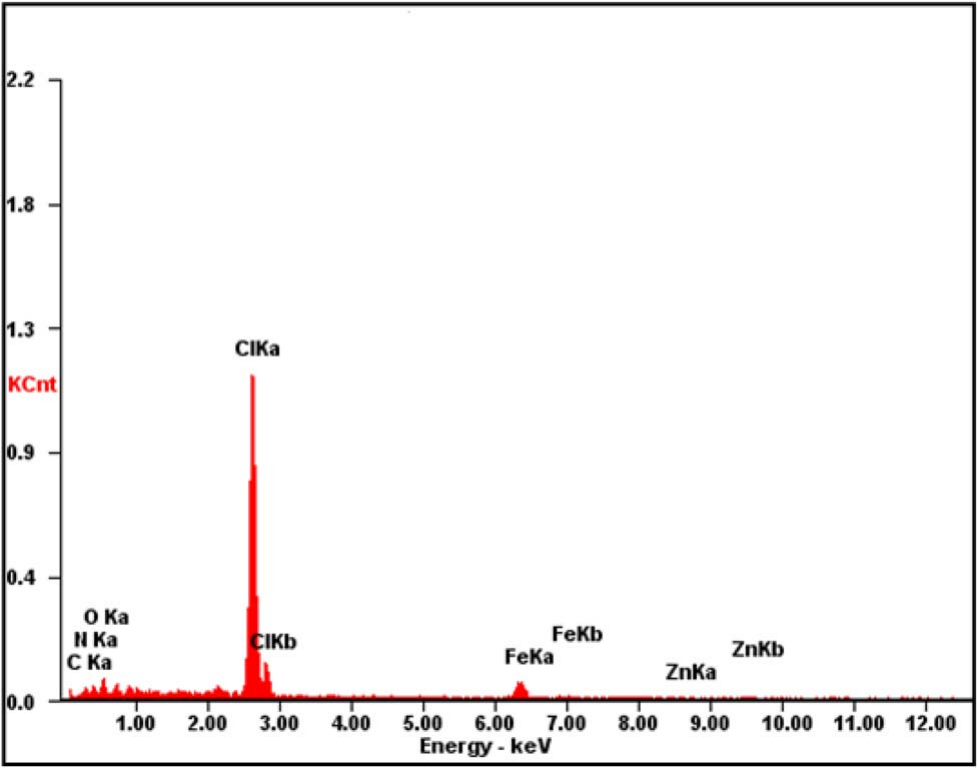
Energy-dispersive X-ray spectrum of CQDs/ZIF-90/PVP/Fe_3_O_4_.

The fabricated CQDs, CQDs/ZIF-90, CQDs/ZIF-90/PVP and CQDs/ZIF-90/PVP/Fe_3_O_4_ were investigated by XRD and the current spectra are shown in Fig. 4. As shown in Fig. 4, a characteristic peak has been located at 2*θ* = 27° which reveals the existence of amorphous carbon structure.^21^ The modification of CQDs with ZIF-90 and the crystallinity of ZIF-90 were investigated by XRD. ZIF-90 showed sharp peaks centered at 2*θ* = 10.26°, 12.62°, 17.48° and 19.87°, revealing that ZIF-90 has a crystalline structure, which is in good accordance with the simulated pattern reported in the literature.^37^ CQDs/ZIF-90/PVP showed a broad peak at 2*θ* = 22.5, revealing the amorphous nature of polyvinyl pyrrolidone.^44^ The XRD pattern of the CQDs/ZIF-90/PVP/Fe_3_O_4_ nanocomposite shows different peaks at 2*θ* = 23.22°, 32.31°, 40.20°, 47.12°, 53.27° and 58.11°, confirming the formation of Fe_3_O_4_. The results indicate the cubic crystal structure due to the sharp and narrow peaks, which is in agreement with the data reported in the literature.^45^Fig. 5 shows the energy-dispersive X-ray (EDX) spectrum of the fabricated nanosorbent. The existence of N, S, Fe, O, Zn and C is confirmed showing the successful synthesis of sorbents.

**Fig. 6:**
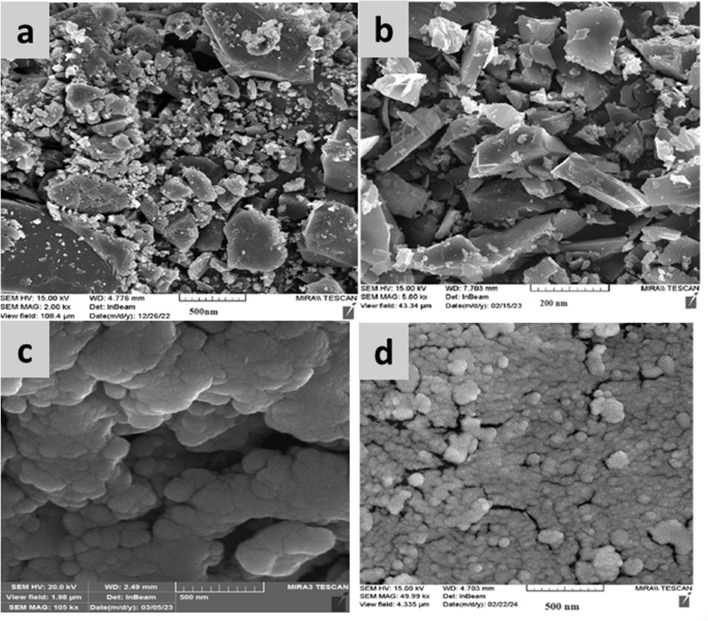
Field-emission scanning electron microscopic (FE-SEM) images of (a) CQDs, (b) CQDs/ZIF-90, (c) CQDs/ZIF-90/PVP and (d) CQDs/ZIF-90/PVP/Fe_3_O_4_.

The morphology and particle size of CQDs, CQDs/ZIF-90, CQDs/ZIF-90/PVP and CQDs/ZIF-90/PVP/Fe_3_O_4_ were studied using FE-SEM images. Fig. 6a shows that the nanosheets of CQDs formed a layered shape with some numbers of residues of –OH and –COOH on the surface of CQDs. Fig. 6b displays the existence of ZIF-90 on the surface of CQDs. The crystalline structure of ZIF-90 with nanoscale size is confirmed. Furthermore, the modification of CQDs/ZIF-90 with PVP was tested by evaluating the FE-SEM image illustrated in Fig. 6c. As it can be seen, the conductive polymer was coated on the surface of the sorbent by a layer structure. The polymer is well dispersed and it may grow on the surface area and provide better performance of sorbents for the enrichment of analytes. In addition, the polymer may avoid the agglomeration of CQDs/ZIF-90 due to the surface functionalities. Fig. 6d shows that Fe_3_O_4_ particles are formed in the CQDs/ZIF-90/PVP nanocomposite with a spherical morphology with notable distribution on the surface.

### 3.2. Evaluation of variables

In this study, the main variables affecting the performance of the present method including sorbent amount, pH, extraction time, ionic strength, volume of desorbing agent and desorption time were evaluated in the monovariate strategy, and the optimum values were selected.

#### 3.2.1. Effect of CQDs/ZIF-90/PVP/Fe_3_O_4_ amount

**Fig. 7:**
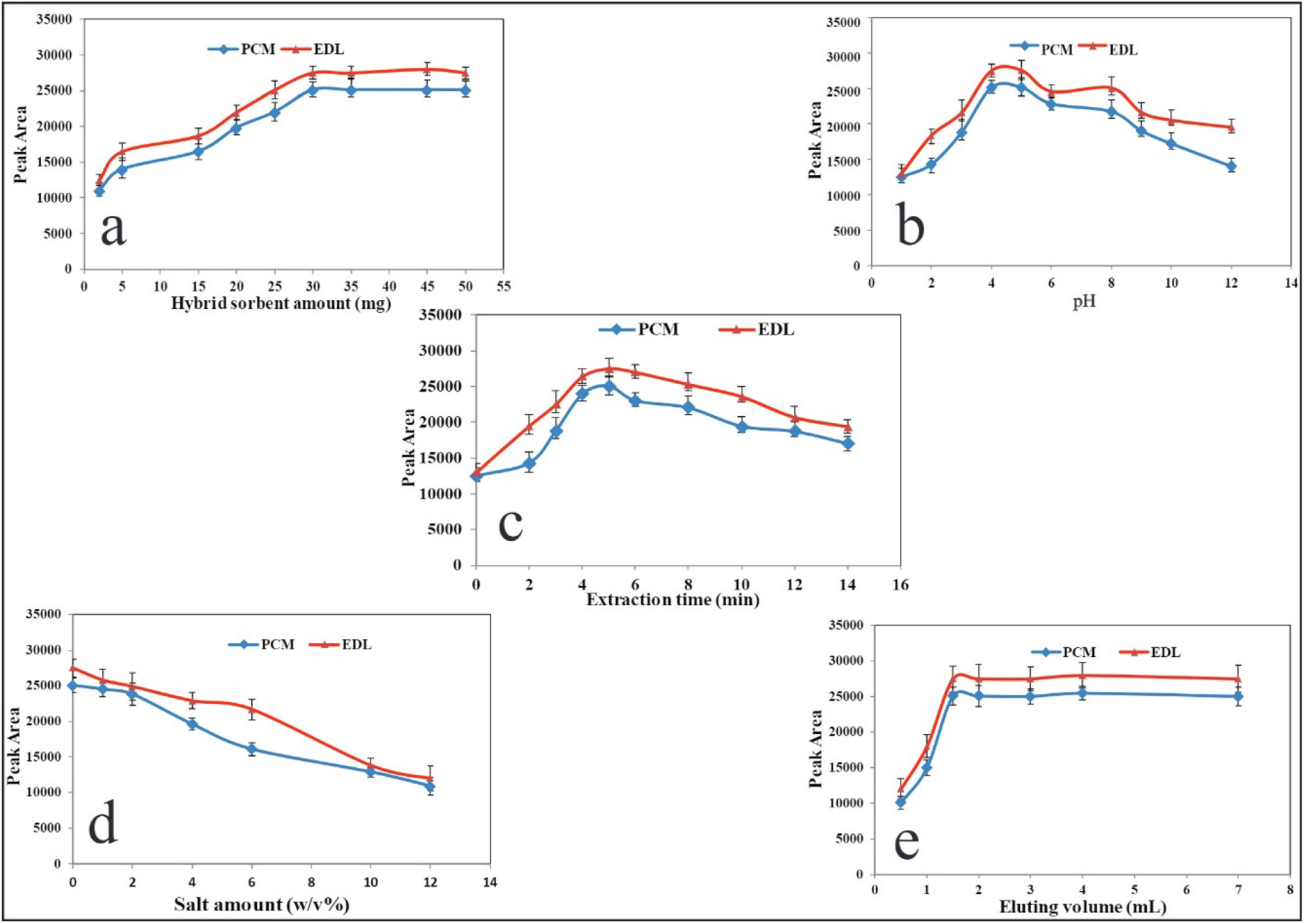
Variables affecting the performance of US-A-MDMSPE-HPLC-UV. Effect of (a) the dosage of CQDs/ZIF-90/PVP/Fe_3_O_4_, (b) pH, (c) extraction time, (d) salt amount and (e) volume of desorbing agents. Other conditions: PCM and EDL at a concentration level of 200 ng mL^−1^ for every drug, sample volume of 5.0 mL, desorption time 4.0 min.

In the micro-solid-phase extraction, the nanosorbent amount plays a significant role in producing reproducible and sensitive data. Furthermore, nano-scale sorbents provide a higher surface area, resulting in efficient extraction and enrichment. To optimize this variable, different amounts of CQDs/ZIF-90/PVP/Fe_3_O_4_ within the range of 1.0–50.0 mg were subjected to the extraction protocols and subsequent data for PCM and EDL were studied. As shown in Fig. 7a, the analytical responses increase as the mass of sorbent grows and they reach the maximum value at 30.0 mg. At higher values of 30.0 mg, the signals reduce slightly due to the agglomeration of sorbent particles and, from a certain value, data remain approximately constant. The most sensitive signals were achieved at a low dosage of nano-sorbent during the short extraction time, which exhibits the considerable porosity of the CQDs/ZIF-90/PVP/Fe_3_O_4_ surface. For subsequent steps, 30.0 mg of nanosorbent was selected as the optimal dose.

#### 3.2.2. Effect of pH

The ionizable compounds are affected by the pH of the sample solution in which the neutral or ionic forms are created depending on the concentration of hydronium in the solution. The pKa values of ionizable compounds play a significant role to push the equilibrium toward the formation of molecular or ionized forms according to the pH of the solution. PCM and EDL are ionizable drugs and their pKa values must be taken into account to understand the effect of pH on the extraction performance. The pKa values of PCM and EDL are 5.3 and 4.6, respectively, so at an acidic pH, the molecular form is prominent.^46,47^ As it can be seen in Fig. 7b, the effect of pH was investigated within the range of 1.0–12.0 using 0.01 M HCl and NaOH, and the highest and reproducible signals were obtained at pH 4.0. The fabricated sorbent reveals the hydrophobic structure, thus at acidic pH, the hydrophobic–hydrophobic interaction between the molecular form of drug and sorbent occurs, resulting in a higher extraction yield. Hence, a pH of 4.0 was selected for the rest of the work.

#### 3.2.3. Effect of extraction time

In this study, ultrasound irradiation was used for the dispersion of nano-scale sorbent throughout the sample media during a certain time. The time of applying ultrasound irradiation as the extraction time is a critical value to result in the complete adsorption of analytes on the surface of sorbents and provide higher sensitivities in data.^48^ Furthermore, this parameter should be optimized to prevent a tedious extraction process. Hence, the ultrasound irradiation was applied from 0 to 14 min, and the results are shown in Fig. 7c. As it is clear, from 0 to 5 min, the signals increased and after that a significant drop was observed. The reduction in extraction yield at a higher time may be due to the return of analytes from the sorbent to the aqueous solution. According to these criteria, the extraction time of 5 min, at a frequency of 25 kHz and a power of 60 W, was selected for the rest of the work.

#### 3.2.4. Effect of ionic strength

It is well documented that the salt content of the sample affects the solubility of compounds in aqueous media.^49^ To evaluate this variable and its effect on the adsorption of drugs under study, the NaCl concentrations within the range of 0–10% W/V were tested. Fig. 7d displays the effect of NaCl concentration on the quantification of PCM and EDL. As the recent results reveal, by increasing the salt contents, the extraction recovery declines, which may be due to the increase in the viscosity of solution causing low analyte mass transfer. As a result, no electrolyte was used for all experiments.

#### 3.2.5. Kind of elution, its volume and desorbing time

In order to elute the analytes from the nanosorbent, some organic solvents including methanol, acetonitrile and acetone were subjected to US-A-MDMSPE. In this evaluation, it was found that among the desorbing solvents, acetonitrile revealed better performance regarding sensitivity and reproducibility of data. In addition, the volume of eluting agent was tested from 0.5 to 7.0, and from the results shown in Fig. 7e, it can be found that 2.0 mL of eluting agent is high enough to desorb PCM and EDL from the extractor and provide stable signals. To evaluate the desorbing time, the elution process through applying ultrasound irradiation was performed from 1 to 8 min. The desorbing time of 4 min is sufficient to provide desirable extraction performance.

### 3.3. Reusability of nanosorbents

In order to present a cost-effective extraction strategy, it is so important that the nanosorbent exhibits a desirable reusability. To investigate the current ability of CQDs/ZIF-90/PVP/Fe_3_O_4_, it was washed with 1.5 mL acetonitrile and 1.5 mL double-distilled water through applying ultrasonic irradiation for 5 min. After the elution of sorbents, it was used again and the obtained results revealed that only 6% decrease in extraction recovery is observed after 12 cycles. The pH range between 1 and 12 and the temperature range from 20 °C to 50 °C were checked, and the stability of the sorbent when it is exposed to the visible light for more than 1 h was tested. Furthermore, only a reduction of 2% in analyte recovery at concentration level of 200 ng mL^−1^ was observed.

### 3.4. Analytical performance

US-A-MDMSPE followed by HPLC-UV provided appreciable analytical figures of merit for the quantification of PCM and EDL, as shown in Table 1. The present features include linear dynamic range (LDR), calibration curve equation, coefficient of determination (*r*^2^), limit of detection (LOD), limit of quantification (LOQ), concentration factor (CF), enrichment factor (EF) and extraction recovery (ER). The CF factor was calculated based on the ratio of initial volume of analyte to final volume of analyte. The FE factor was measured using the ratio of the slopes of the calibration curves before and after the extraction method. Practical linear dynamic ranges from 0.7 to 2000 ng mL^−1^ and 0.5 to 1200 ng mL^−1^ were achieved for PCM and EDL with appreciable linearity within 0.993 to 0.998, respectively. The LODs for PCM and EDL were 0.16 ng mL^−1^ and 0.09 ng mL^−1^, respectively. As shown in Table 1, the analytical features of the present method meet all the requirements for the quantification of the current drugs in real samples. ER was calculated based on the following equation:ER% = EF × (*V*_Final volume_/*V*_Initial volume of plasma_) × 100

**Table 1:** Analytical features of US-A-MDMSPE-HPLC-UV in the determination of paracetamol and etodolac

Analyte	LR^a^ (ng mL^−1^)	Linear equation	(*r*^2^)^b^	LOD^c^ (ng mL^−1^)	LOQ^d^ (ng mL^−1^)	CF^e^	EF^f^ %	ER^g^ % (*n* = 3)
Paracetamol	0.7–2000	*Y* = 125*X* + 76	0.993	0.16	0.7	40	35.6	89.1
Etodolac	0.5–1200	*Y* = 137*X* + 91	0.998	0.09	0.5	40	37.4	93.5

a Linear range (LR).

b Correlation coefficient (*r*^2^).

c Limit of detection (LOD).

d Limit of quantification (LOQ).

e Concentration factor (CF).

f Enrichment factor (EF) calculated for 200 ng mL^−1^ of each drug.

g Extraction recovery (ER).

**Fig. 8:**
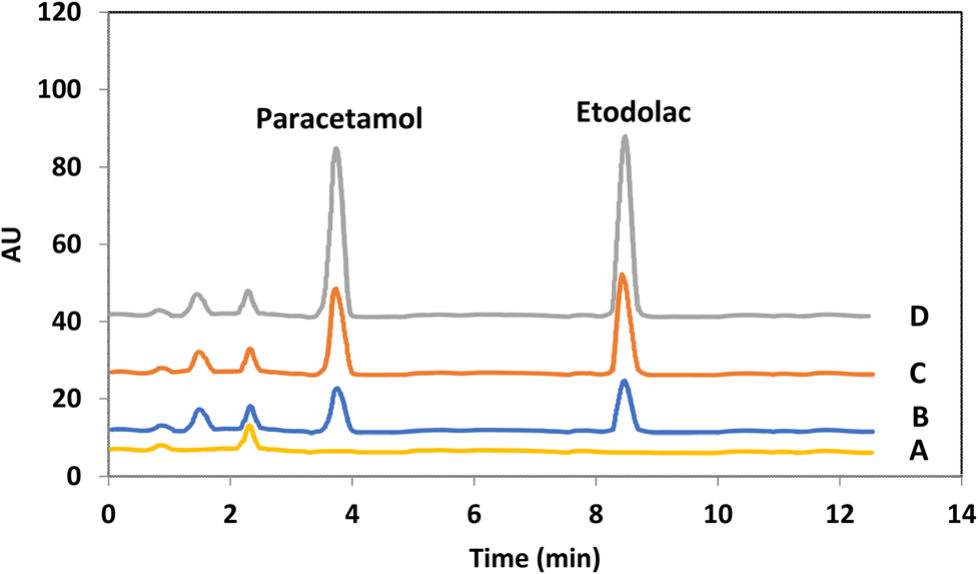
Different HPLC–UV chromatograms of the target drugs of PCM and EDL in human plasma: (A) blank; human plasma samples spiked with the concentration level of each drug at (B) 100 ng mL^−1^, (C) 200 ng mL^−1^ and (D) 400 ng mL^−1^.

Fig. 8 shows the HPLC chromatograms of the blank sample and spiked sample with different levels of drugs. As it is clear after applying the extraction procedure, the compounds which usually exist in the plasma sample reveal no notable effect on the determination of target drugs.

### 3.5. Evaluation of precision and accuracy

Different QC samples for each drug at concentration levels of 10, 250, 500 and 1500 ng mL^−1^ were subjected to US-A-MDMSPE followed by HPLC-UV for quantification to evaluate the precision and accuracy of the method. For the latter goals, intra-assay (with 1 day) and inter-assay (with 3 days) precisions and accuracies should be taken into account. Intra-day and inter-day experiments were conducted and relative standard deviations (RSDs) as precision factors were studied and evaluated for PCM and EDL. Relative errors in every term of analysis were calculated as the accuracy parameter. As Table 2 summarizes, all the mentioned data, appreciable accuracies (≤8.5%) and notable intra-assay (≤6.9%) and inter-assay (≤8.3%) precisions exhibit the applicability of the method for the determination of target drugs in trace levels in real media.

**Table 2:** Evaluation of intra-day and inter-day accuracy and precision results for the quantification of paracetamol and etodolac

Drug	Concentration (ng mL^−1^)	Intra-day, *n* = 12				Inter-day, *n* = 12	
		Found value ± SD^a^,^b^ (ng mL^−1^)	RSD^c^ (%)	Accuracy^d^ (%)	Found value ± SD (ng mL^−1^)	RSD (%)	Accuracy (%)
Paracetamol	Low QC (10)	9.6 ± 0.5	5.2	−4.0	9.2 ± 0.7	7.6	−8.0
	Medium QC 1 (250)	271.2 ± 14.7	5.4	+8.5	229.0 ± 17.0	7.4	−8.4
	Medium QC 2 (500)	480.2 ± 30.7	6.4	−4.0	526.6 ± 38.2	7.2	+5.3
	High QC (1500)	1428.9 ± 75.1	5.2	−4.7	1439.0 ± 80.1	5.6	−4.0
Etodolac	Low QC (10)	9.3 ± 0.6	6.4	−7.0	9.2 ± 0.6	6.5	−8.0
	Medium QC 1 (250)	231.4 ± 11.6	5.0	−7.4	230.1 ± 15.5	6.7	−7.7
	Medium QC 2 (500)	519.7 ± 36.0	6.9	+3.9	482.35 ± 40.1	8.3	−3.6
	High QC (1500)	1567.1 ± 90.8	5.8	+4.5	1583.2 ± 97.7	6.2	5.5

a Standard deviation.

b The mean of three independent analyses.

c It was defined according to the following equation: 100 × SD/mean.

d It was calculated based on the following equation: 100 × (mean concentration found − known concentration)/(known concentration).

### 3.6. Pharmacokinetic assessment

In order to test the applicability of the method for qualification, PCM and EDL were analyzed in human plasma. For the correct purpose, six healthy volunteers aged 25–33 years took a tablet containing combined doses (500 mg PCM/400 mg EDL) by oral administration. All the volunteers were asked to avoid alcoholic drinks or cigarette until sampling. After each volunteer received the drug, the blood samples were collected on days 0, 1, 2, 3, 4, 6, 8, 10 and 12. Every sample was transferred to the tube containing EDTA, centrifuged at 5000 rpm for 5 min and stored at −18 °C. For the evaluation of pharmacokinetic data including maximum plasma concentration (*C*_max_), half-life (*T*_1/2_), area under the curve from zero to the last sampling time (AuC_0–12_) and area under the curve from zero to infinite time (Acu_0–∞_), plasma samples were analyzed *via* the present method and the average concentrations of each drug *versus* sampling time were plotted. All the pharmacokinetic parameters are reported in Table 3. From the data summarized in this Table, it can be found that the application of US-A-MDMSPE prior to HPLC-UV is a reliable and alternative protocol for the quantitation of PCM and EDL in human plasma after combination therapy.

**Table 3:** Pharmacokinetic evaluation of paracetamol and etodolac after oral administration of the tablet (500 mg paracetamol/400 mg etodolac) to six healthy volunteers^a^

Pharmacokinetic feature	Mean ± SD	
	Paracetamol	Etodolac
*T*_max_ (h)	1.21 ± 0.40	1.56 ± 0.61
*C*_max_ (μg mL^−1^)	13.12 ± 0.90	28.65 ± 4.20
AUC_0–12_ (μg h mL^−1^)	47.91 ± 3.25	115.70 ± 7.48
AUC_0–∞_ (μg h mL^−1^)	56.61 ± 4.75	153.9 ± 14.75
*T*_1/2_ (h)	1.80 ± 0.12	7.23 ± 0.45

a *T*_max_: Time required for obtaining maximum concentration; *C*_max_: maximum target drug concentration in plasma; AUC _0–12_: area under the curve; AUC _0–∞_: area under the curve at an infinite period of time; *T*_1/2_ (h): time required for obtaining half concentrations of each drug.

### 3.7. Comparison with other methods

The main analytical figures of merits of the present method were compared with other methods for the quantification of PCM and EDL (Table 4). As it can be seen, there was a significant improvement in LODs and LOQs, which makes the method more practical for quantitative features. In addition, reasonable RSD values and comparable extraction times as well as acceptable cost of analysis demonstrated the present US-A-MDMSPE method followed by HPLC-UV can be considered as an alternative approach for multi-purpose analytical aspects. Furthermore, some of the previously reported techniques suffer from many limitations including low sensitivity, matrix effect, high extraction time, lack of the ability of simultaneous analysis, and tedious steps. As it is clear, *via* the modification of CQDs with ZIF-90, polymer and Fe_3_O_4_, the present extraction protocol overcomes the mentioned limitations and reveals significant advantages over the methods reported in the literature. These advantages are due to the following improvements: high porosity of sorbents, reasonable adsorption capacity, desirable reusability of extractors, matrix removal and reproducibility of data.

**Table 4:** Comparison of US-A-MDMSPE-HPLC-UV with other methods reported in the literature for the determination of PCM and EDL

Detection system	Extraction method	Drug	LOD (μg mL^−1^)	LOQ (μg mL^−1^)	*r* ^2^	RSD (%)	Matrix	LR^k^ (μg mL^−1^)	Refs
RP-HPLC^a^	LLE^b^	EDL	0.03	0.1	0.9998	0.4	Plasma	10–50	50
LC/MS/MS^c^	Large-volume direct injection	PCM	0.000005	0.00001	0.998	8.0–10.0	Water	0.0005–0.5	51
UV	—	EDL-PCM	0.1593–0.0488	0.4827–0.1480	0.9980–0.9984	0.324–0.122	Tablet	2–14, 2–14	52
HPLC-DAD^d^	LLE	EDL	0.918	2.783	0.9995	≤7.58	Rat plasma	5–50	53
GC-MS^e^	SPE^f^	PCM	0.0002–0.0056	NA	0.998–0.999	≤10	Blood/urine	0.0006–5	54
RP-HPLC	LLE	EDL-PCM	0.33–0.36	1.01–1.09	0.9994–0.9983	0.9–0.7	Pharmaceutical dosage form	3–16, 5–25	55
HPLC-PDA^g^	SBC-18SPE^h^	PCM	0.2	0.8	0.9987	3.45	Tablet	0.8–270	56
RP-HPLC	One step LLE	EDL	—	0.1	0.99	20	Human plasma	0.1–25	57
LC/MS/MS	SBSE^i^	PCM	0.000005–0.00001	0.00002–0.00004	NR	10.10–17.0	Water	2–200	58
RP-HPLC	M-MSPD^j^	PCM	0.008	0.024	—	≤2.02	Wastewater	—	59
RP-HPLC	US-A-MDMSPE	PCM-EDL	0.00016–0.00009	0.0007–0.0005	0.993–0.998	≤7.6–≤8.3	Human plasma	0.0007–2, 0.0005–1.2	This work

a RP-HPLC: Reversed-phase high-performance liquid chromatography.

b LLE: Liquid–liquid extraction.

c LC/MS/MS: Liquid chromatography tandem mass spectrometry.

d HPLC-DAD: High-performance liquid chromatography–diode array detection.

e GC-MS: Gas chromatography-mass spectrometry.

f SPE: Solid-phase extraction.

g HPLC-PDA: High-performance liquid chromatography-photometric diode array.

h SBC-18SPE: Silica-based C-18 solid phase extraction.

i SBSE: Stir bar sorptive extraction.

j M-MSPD: Magnetic matrix solid-phase dispersion.

k Linear range (LR).

## 4. Conclusion

A promising four-part and recyclable non-absorbent based on CQDs immobilized with ZIF-90, polyvinyl pyrrolidone and Fe_3_O_4_ was fabricated and used as a new and alternative extractor in US-A-MDMSPE. The designed extraction protocol was applied for the isolation and enrichment of PCM and EDL prior to HPLC measurements. The modification of CQDs with special substances offers many benefits including reasonable aromatic–aromatic interactions, appreciable mass transfer, adjustable porosity of sorbents, easy recyclability of the four-part nanosorbent from aqueous media using a magnet, applicable reusability, and increased area-to-volume ratio. The successful pharmacokinetic assessments with low-dose administration of both target drugs through the combination therapy demonstrated that the nanoporous sorbent in combination with the chromatography tool is a convincing protocol for screening purposes and bioequivalence evaluations.

## Data availability

All data are provided in this manuscript.

## Author contributions

All authors have agreed to their individual contributions ahead of submission.

## Conflicts of interest

There are no conflicts to declare.

## Supplementary Material

RA-014-D4RA04875J-s001
