# Supplementary material for: Ultrasound-assisted magnetic dispersive micro-solid-phase extraction based on carbon quantum dots/zeolite imidazolate framework-90/polyvinyl pyrrolidone/Fe3O4 followed by high-performance liquid chromatography with ultraviolet detection for trace analysis of paracetamol and etodolac in human plasma
Source: RSC Adv. 2024 Oct 22;14(45):33418–28. doi: 10.1039/d4ra04875j (PMC11495277; doi:10.1039/d4ra04875j)
Supplement: RA-014-D4RA04875J-s001 [file RA-014-D4RA04875J-s001.pdf]

Supplementary Information (SI) for RSC Advances.
This journal is © The Royal Society of Chemistry 2024

Supporting information

Ultrasound-assisted magnetic dispersive micro-solid phase extraction based on carbon quantum dots/zeolite imidazolate framework-90/polyvinyl pyrrolidone combined with high performance liquid chromatography with ultraviolet detection for trace analysis of paracetamol and etodolac in human plasma

Kimia Ahmadi[1], Aysan Abolfathi[1], Sana Nasirimoghadam[1], Negar Nasiri Moghaddam Kalkhoran[1], Mohsen Zeeb[*,1]

[1] *Department of Applied Chemistry, Faculty of Science, South Tehran Branch, Islamic Azad University, Tehran, Iran*

[*] Corresponding author, e-mail address: zeeb.mohsen@gmail.com and Tel.: + 98 21 33722831.

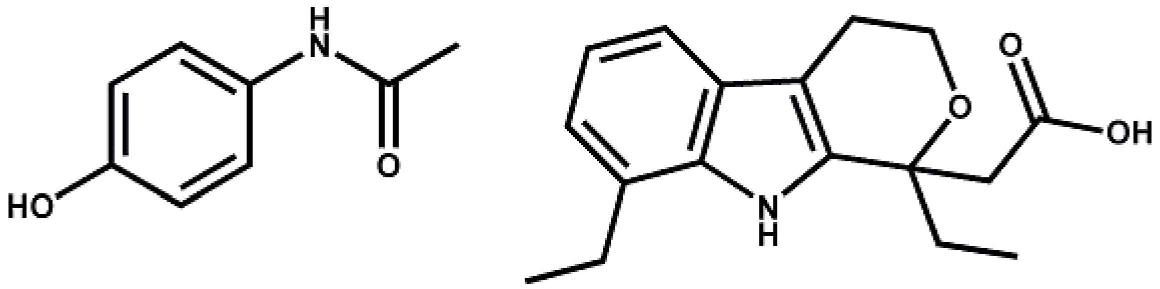

A B

Fig 1S. Chemical structures of drugs: (A) PCM (Chemical Formula: $C_8H_9NO_2$, MW:151.16 g/mol), (B) EDL (Chemical Formula: $C_{17}H_{21}NO_3$, MW: 287.37 g/mol)
